# Ferromagnetic-like behavior of Co doped TiO_2_ flexible thin films fabricated via co-sputtering for spintronic applications

**DOI:** 10.1016/j.heliyon.2020.e03338

**Published:** 2020-02-08

**Authors:** Heiddy P. Quiroz, E.F. Galíndez, A. Dussan

**Affiliations:** Universidad Nacional de Colombia - Bogotá, Department of Physics, Grupo de Materiales Nanoestructurados y sus Aplicaciones, Cra. 30 No. 45-03 Edificio 404 Yu Takeuchi Lab. 121C / 121B-1 Ciudad Universitaria – Bogotá, 11001, Colombia

**Keywords:** Condensed matter physics, Materials chemistry, Materials science, Ferromagnetic-like, Titania, Dipolar interaction, Flexible substrate

## Abstract

TiO_2_:Co thin films on ITO (Indium-tin-oxide)/PET (Poly Ethylene Terephthalate) flexible and glass substrates were fabricated via DC magnetron co-sputtering at room temperature. The samples deposited on glass substrates were subjected to annealing processes at 473 K for 2 h to improve the crystallization of the material. Both TiO_2_:Co/ITO/PET and TiO_2_:Co/glass thin films exhibited excellent optical properties with more than 80% transmission in the visible region. An increase on ITO/PET surface temperature was detected during the synthesis of the samples; this variation in the ITO substrate (∼ 2 K) was associated to impact energetic of ion or atoms bombarded during the deposition process. X-ray diffraction measurements evidenced local phases to growth on the flexible substrate; the random distributions of Cobalt crystals into the rutile and anatase phases were associated to a crystalline lattice embedded with magnetic ions. A configuration of small grains and absence of cluster formation on the surface of thin films was observed through SEM and AFM measurements. From this topographic study and MFM measurements evidenced that surface grains were not constituted magnetic domains formation in the thin films. The ferromagnetic-like behavior was observed in a magnetization as function of field measurement by PPMS. M vs H curves at room temperature for TiO_2_:Co/ITO/PET thin films, showed the hysteresis loop. The dipolar interaction between Cobalt ions without formation of domains were correlated to the magnetic behavior in the material, as the doping concentration is lower than 12%.

## Teaser

Condensed matter physics; Materials chemistry; Materials science; Ferromagnetic-like.; Titania; Dipolar interaction; PET.

## 1 Introduction

In recent years, development of flexible electronic devices has been studied because to the difficulty of the degradation quality at high temperatures and fabrication method depending on the organic or inorganic material [1]. In particular, polyethylene terephthalate or PET (C_10_H_8_O_4_)_n_ is a polymer material used as substrate for different applications due its properties, such as mechanical strength, inertness to chemical action, transparent condition, among other [2, 3]; this polymer is characteristically opaque, white or transparent depending on whether it is semi-crystalline or amorphous structure [3].

On the other hand, TiO_2_ or Titania is a semiconductor that has attracted attention due to its properties and applications in optical sensors [4], biosensors [5], solar cells [6, 7], magneto-optic [8, 9, 10], and spintronic materials (TiO_2_-doped with transition elements (Mn, Ni, Co)) [11, 12] and non-volatile memories devices (NVM), specifically, resistive random-access memory (RRAM) [13, 14, 15]. However it is well known that, Co-doped TiO_2_ is a ferromagnetic material at room temperature [16]; these properties turn it into a promising material for magnetoresistive random-access memory (MRAM) and spintronic applications. For doped TiO_2_ synthesis, different methods have been implemented such as template sol-gel method [12], RF magnetron sputtering [17], molten-salt method [18], Czochralski growth (CZ) method [19], etc. These synthesis methods have been used to obtain different types of nanostructure formations such as nanoparticles, crystals, thin films, among others.

This work presents a study of the synthesis parameters and morphological, structural, and magnetic properties of TiO_2_: Co thin films deposited via DC magnetron co-sputtering on ITO/PET flexible and glass substrates at room temperature. Co-doped TiO_2_ thin films fabricated on ITO/PET and glass were characterized through SEM, XRD, MFM, and PPMS measurements. Magnetization behavior as a function of applied field and temperature of TiO_2_:Co thin films evidencing the hysteresis loop with low coercive field. The FC (Field Cooling) and ZFC (Zero Field Cooling) measurements were realized and showed a paramagnetic behavior in the thin films at high temperature (>70 K). A correlation between the synthesis parameters and physical properties of the material is presented.

## 2 Experimental procedures

TiO_2_:Co thin films were deposited via DC magnetron co-sputtering on ITO/PET and glass soda lime type substrates at environmental temperature. The thickness of the ITO film is 24 nm and PET substrate is 127 μm thick [20]. TiO_2_ and Co targets of 762 mm diameter, 3 mm thick and 99.9% purity, were used for the synthesis of the samples. Parameters target power (TiO_2_ - 120 W and Co - 25 W), working pressure ($2.5\times 10^{-2}$ Torr), deposition time t_d_ (30 min) and substrate-target distance (70 mm) remained constant. To achieve clean conditions of the internal chamber (absence of particles or oxygen atoms) during the synthesis processes, first a purging process with nitrogen gas and then high vacuum conditions were carried out, to finish the vacuum conditions with established of the work pressure.

**Figure 1:**
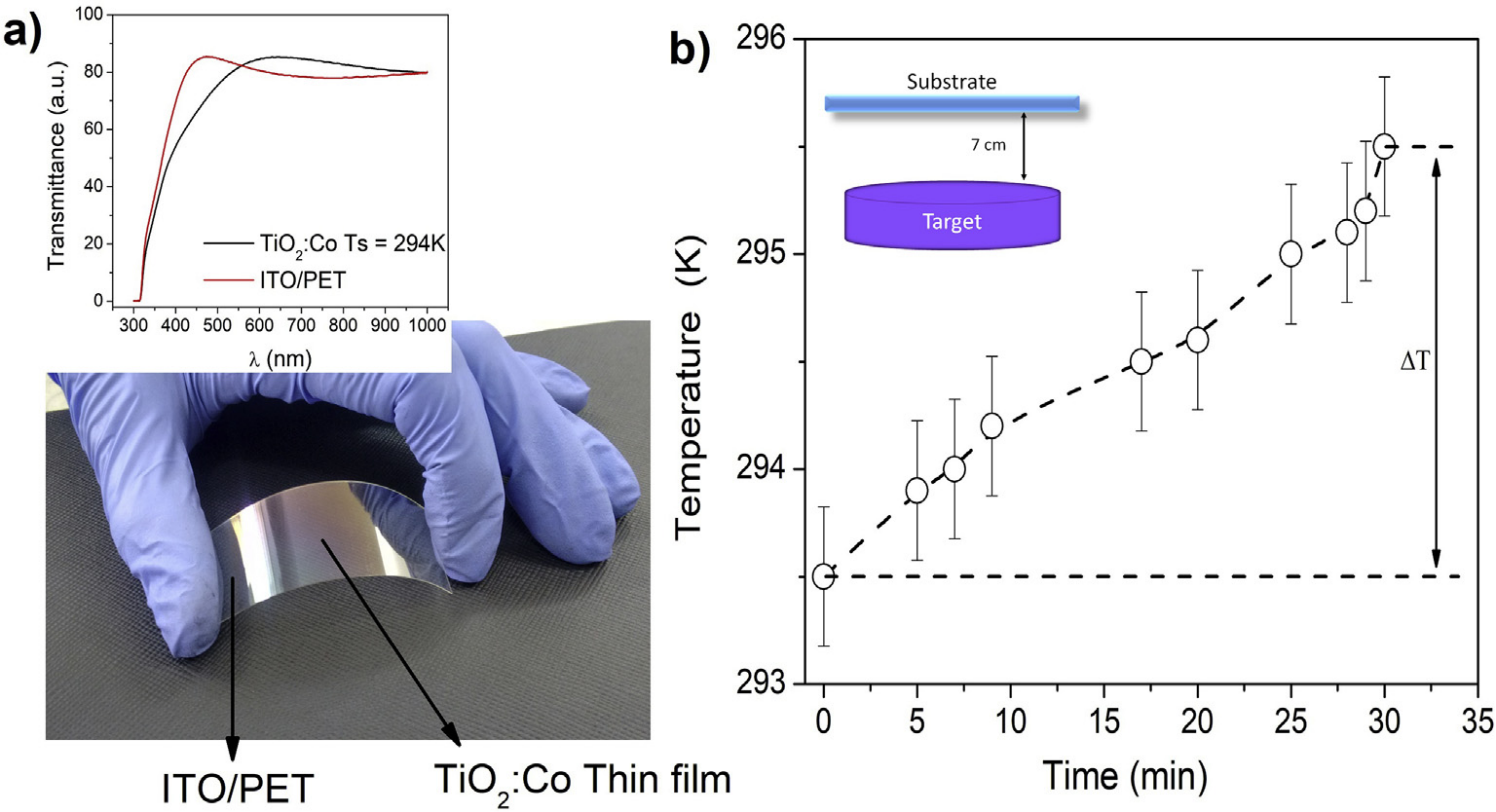
a) Photograph of a TiO_2_:Co thin film on ITO/PET and transmittance spectra of the thin film and ITO/PET substrate (Ts: substrate temperature), and b) substrate temperature as a function of time during deposition of the TiO_2_:Co thin film on ITO/PET.

It is known, in sputtering processes, that the ions of the gas (Ar) that generate the plasma have enough energy to pull atoms off the target by moment transfer. In this case, most of the energy provided by the incident ions is transformed into heat, transferred to the system and generates a slight temperature increase of the PET, contributing to the nucleation process of the atoms that reach the substrate. The effect of the temperature change on the ITO/PET substrate during synthesis is shown in Figure 1b. Some samples on glass substrate were subjected to *in-situ* annealing processes at Ta = 473 K. Figure 1a shows a photograph of TiO_2_:Co thin film on ITO/PET substrate.

The samples were structurally characterized through XRD measurements by using X-ray diffractometer X'Pert Pro polycrystal of PANalytical, equipped with a Cu–K α source: 1.540598 Å, a potential difference of 40 kV, current of 40 mA and X'Celerator detector. The software used for comparison was X'Pert HighScore Plus by Rietveld refinement. μXRD measurements were carried out by using an EMPYREAN diffractometer by PANalytical with Pixcel 3D 2 × 2 detector of high speed and spot of 50 μm × 50 μm, and 40 nm of depth in the range of 10°–90°. The software used for sample analysis was the Microdiffraction Spinner.

Morphological characterization of the samples was used a scanning electron microscope (SEM) Vega3 SB with tungsten source, a detector XFlash Detector 410 M and an acceleration voltage of 10 kV under high vacuum (~10^−6^ mbar) and it have a SDD by EDXS microanalysis; additionally, the domain-structure investigation was carried out by using MFM/AFM modes on the surface of the sample using an Asylum Research MFP 3D Bio in tapping mode with Co tip. This measurement allows both the topographic and the magnetic-force image to be collected separately in the same scan area. Whilst the magnetic properties of the samples were obtained through the VersaLab Magnetic Properties Measurement System (Quantum Design) based on the vibrating sample magnetometer (VSM), complementing the results of the magnetic atomic force microscopy module (MFM) with cobalt tips. The magnetic measurements were investigated in the temperature range of 150 K e 350 K with an applied magnetic field up to 30000 Oe (max.) For each magnetic measurement, the sample was demagnetized by oscillating fields at 300 K to remove the remainder magnetic flux.

Optical properties of the samples were obtained using a spectrophotometer reference T70 + UV/VIS from PG instruments at atmospheric pressure and room temperature.

## 3 Results and analysis

**Figure 2:**
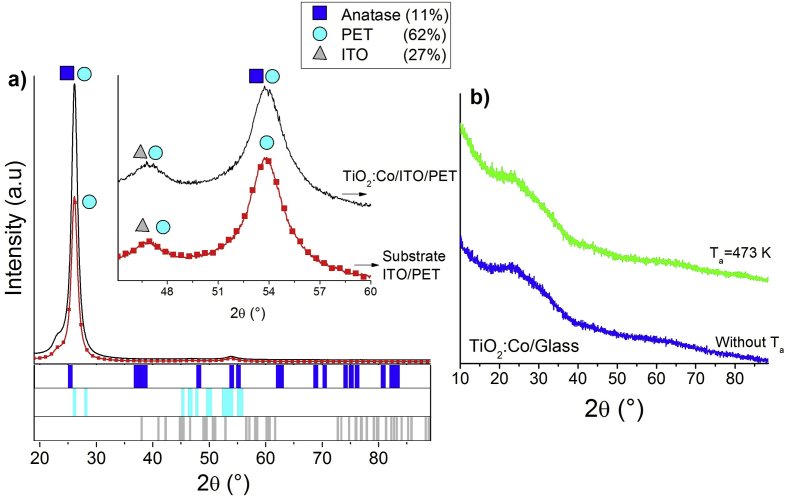
Powder X-ray diffraction pattern for a) TiO_2_:Co/ITO/PET with t_d_ = 30 min and ITO/PET substrate and b) pattern of the thin films on glass without annealing and in situ annealing temperature of 473 K. Inset in Figure 2a shows a zoom pattern at 40 < 2θ < 60.

Figure 2 presents the XRD pattern of TiO_2_:Co thin films on ITO/PET and glass substrates for a deposition time of 30 min and substrate temperature (Ts) of 293.5 K The samples on glass were annealed in situ at 473 K for 2 h; this process was carried out to observe the changes in the micro-crystallinity of the samples, given their amorphous characteristic when they were deposited (blue line, Figure 2b). Even when the XRD measures did not show a notable change in the structure, the thermal energy applied, generate mobility in the chemical species into the thin film. It was observed that the substrate affects the crystallization of the compound on the surface; glass substrate thin films presented an amorphous phase, before and after annealing process; to evidence crystallization processes of TiO_2_ thin films on non-crystalline substrates, annealing stages at high temperatures must be carried out (T ≥ 975 K) [21,222]. On the other hand, TiO_2_:Co thin films on ITO/PET substrate presented the formation of anatase phase (PDF 01-083-2243) (Figure 2a).

The principal diffraction peak in Figure 2a (red line - 2θ = 26.118) characterizes the PET substrate with polycrystalline phase. TiO_2_:Co/ITO/PET thin film shows an overlapping of the diffraction peak with another phases of the ITO/PET substrate. These phases are distinguishable only by widening the peak with respect to the reference pattern of the polymer (Figure 2a). Figure 2b shows the amorphous characteristic of the TiO_2_:Co thin films on glass at both cases, without and in-situ annealing temperature of 473 K.

**Figure 3:**
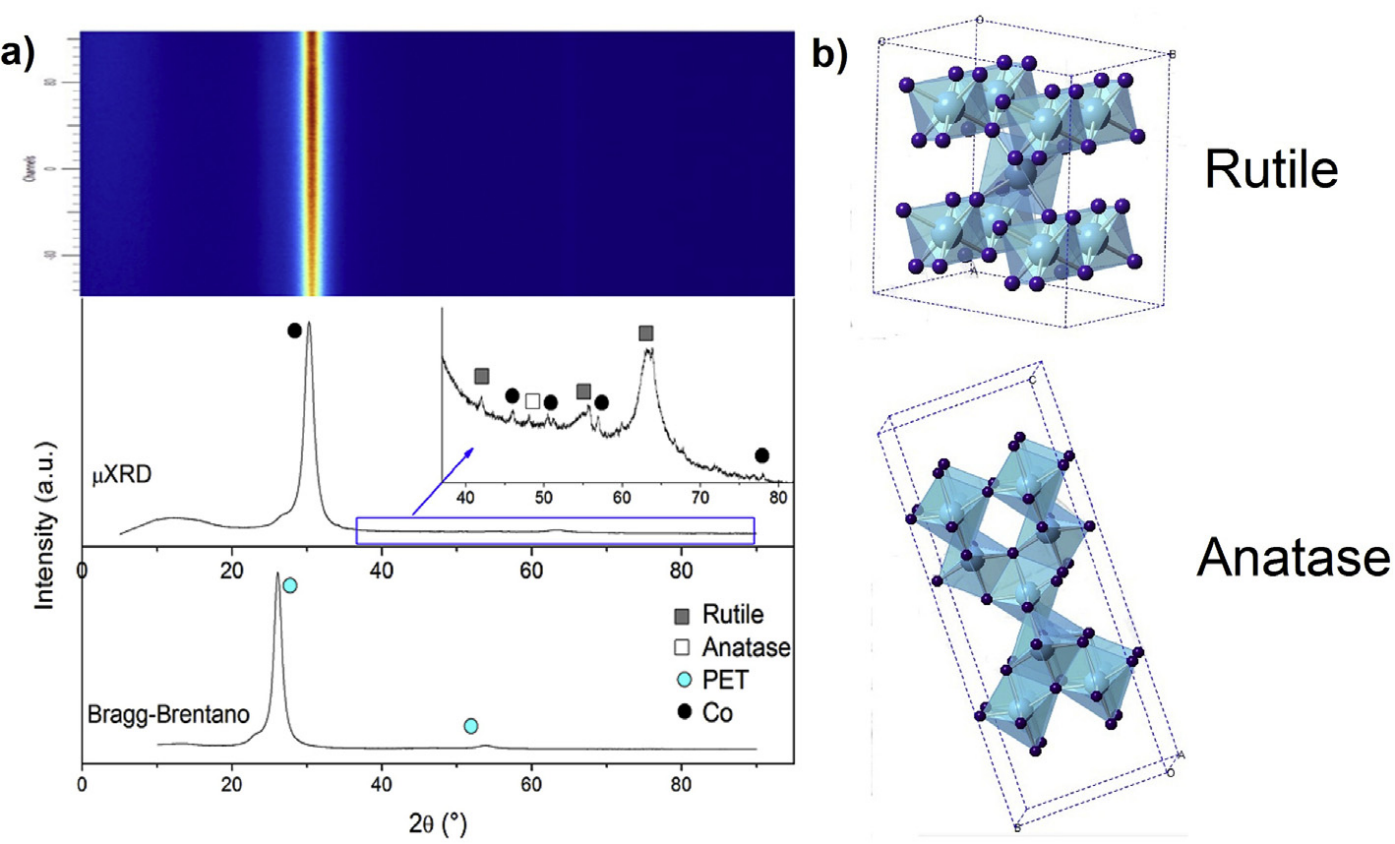
a) Debye rings, μXRD (upper) and XRD (lower) measurements of a TiO_2_:Co/ITO/PET thin film, and b) Crystalline structure of anatase and Rutile phases.

Figure 3a shows a comparison between the Bragg-Brentano configuration and X-ray microdiffraction (μXRD) of a TiO_2_:Co/ITO/PET thin film. μXRD evidences the formation of Co crystals and TiO_2_ binary phases of rutile (PDF 01-076-0324) and anatase (PDF 01-083-2243) (Figure 3b). These phases are only observed through μXRD possibly due to their segregated nature. Nevertheless, due these crystallization planes are not founded in all materials, their contributions are less on glass than on ITO/PET substrates. This is evident in the Debye rings (Upper Figure 3a), where the principal peak corresponds to the Co crystal.

Through Rietveld refinement of the μXRD measurement, quantification of the local phases in the TiO_2_:Co/ITO/PET thin film founded 64.3% Co and 35.7% TiO_2_ polymorphous. These results agree with the formation of smaller crystals of Co in the TiO_2_ semiconductor matrix. This is possible to associate to a segregated location of smaller crystals of Co in the TiO_2_ semiconductor matrix due possibly to the species mobility by diffusion in the deposit process and the thermal stabilization of TiO_2_.

The formation of TiO_2_ is characterized by the synthesis temperature and substrate type [23, 24]. When synthesis temperature was increased, changes in the octahedral TiO_2_ occurred, allowing the formation of anatase and rutile phases; whilst Co ions can be introduced into the TiO_2_ structure by diffusion (diffusion coefficient ~ $1.9\times 10^{-6}m^{2}/s$ [25]), stability of the octahedral rutile and anatase structures, during to the deposit and the annealing process, permitting the Co random distribution into the semiconductor matrix. Therefore, the TiO_2_:Co thin film deposited on ITO/PET evidenced the formation of segregated Co crystals in the sample and the thin film deposited on glass did not evidence the formation of this segregated crystal.

The formation of the Co crystals into the TiO_2_/ITO/PET samples according to the Ti–O system thermal stability. This system establishes the temperature and pressure required to have TiO_2_ in a metastable structure [26, 27]. The changes in the temperature (due to deposit and annealing processes) modified the octahedral structure permitting the polymorphous forms (rutile, anatase and brookite) and Co diffusion [26, 28, 29, 30, 31, 32]. However, the inclusion of ions with a lower valence state into the TiO_2_ matrix (Co^2+^ and Co^3+^ in this case), increased the TiO_2_ crystallization and anatase to rutile transition [26].

In the case of Co, secondary phases or Co oxide were not found, according to the XRD patterns. This oxides formation required the two oxidation state of Co [33] and higher proportion in comparison to the oxygen presence [33]. The low Co concentration (~12 wt% for the all samples) and growth conditions permitting the formation of the diluted TiO_2_:Co thin film when Ts = 293.5 K and Ta = 473 K.

**Figure 4:**
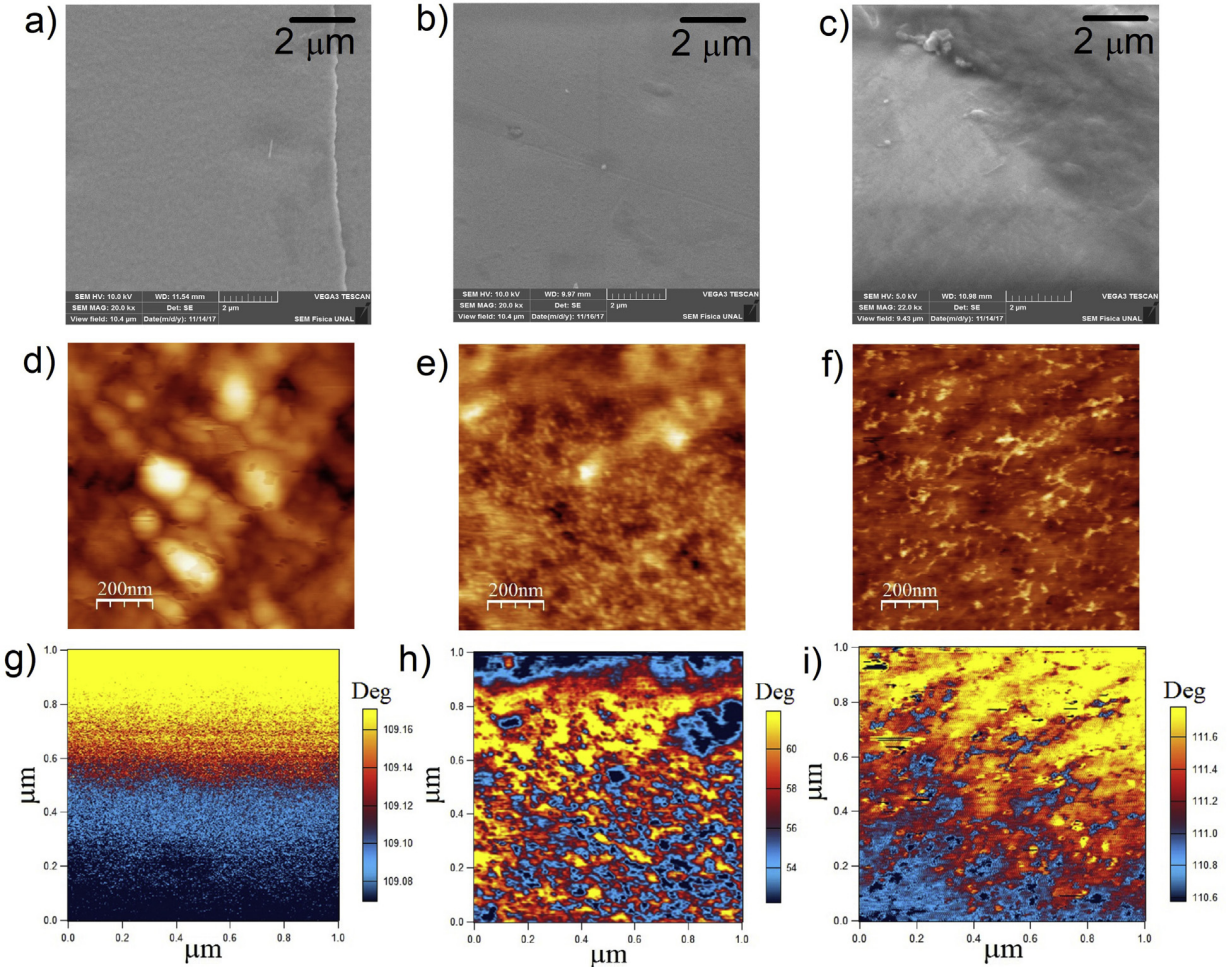
SEM micrographs of thin films: a) Ts = 293.5 K on ITO/PET substrate, b) Ts = 293.5 K on glass substrate, c) Ta = 473 K on glass substrate, AFM and MFM micrographs of TiO_2_:Co thin films on d), g) ITO/PET, e), h) glass substrates without Ta, and f), i) glass substrates with Ta = 473 K.

Figure 4 shows the SEM, AFM, and MFM micrographs of thin films on several substrate types. Thin film growth via DC magnetron co-sputtering is characterized by steps or zones depending of the substrate temperature (Ts) and melting temperature of targets (Tm) (MD model) [21]. This model establishes the growth structure of thin films in zones, where the T zone or transition zone is characterized by very small and elongated grains due to the superficial diffusion contributing to the species mobility among grains [34].

From SEM images (Figures 4a, 4b, 4c), it is possible to observe that thin film surface is governed by smaller grain formation resulting in continuous and homogenous layers of the material. According to the MD model, the T zone happens when the relation between the substrate temperature and melting temperature of the targets is between 0.1 < Ts/Tm < 0.3. Considering that the melting temperature of the TiO_2_ target is 2103 K and the Co target is 1768 K, the Ts/Tm relationship obtained was 0.14 and 0.16, respectively. During the deposition process, the substrate temperature increased from 293.5 to 295.5 K; it can be associated to the increased kinetic energy of the incident species on the substrate. Therefore, at low ITO/PET substrate temperature, superficial diffusion of the Co species and small grains on the surface prevail (Fig. 4d, e, f).

ITO/PET sample surface morphology (Figures 4d, 4g) was characterized by a not homogeneous grain size and roughness mean square of 5.22 (5) nm; whiles the MFM micrographs showed magnetic regions organized in a homogeneous fold type without contribution from the topographic signal. TiO_2_:Co/glass thin film presented a variation in the direction of the magnetic orientation. This was modeled by the presence of oriented, random and not delimited magnetic moments induced by the Co ions into the samples.

On the other hand, the samples deposited on glass substrates, annealed (Figures 4f, 4i) and without annealed (Figures 4e, 4h), showed a magnetic surface distinguished by a non-presence of magnetic domains.

**Figure 5:**
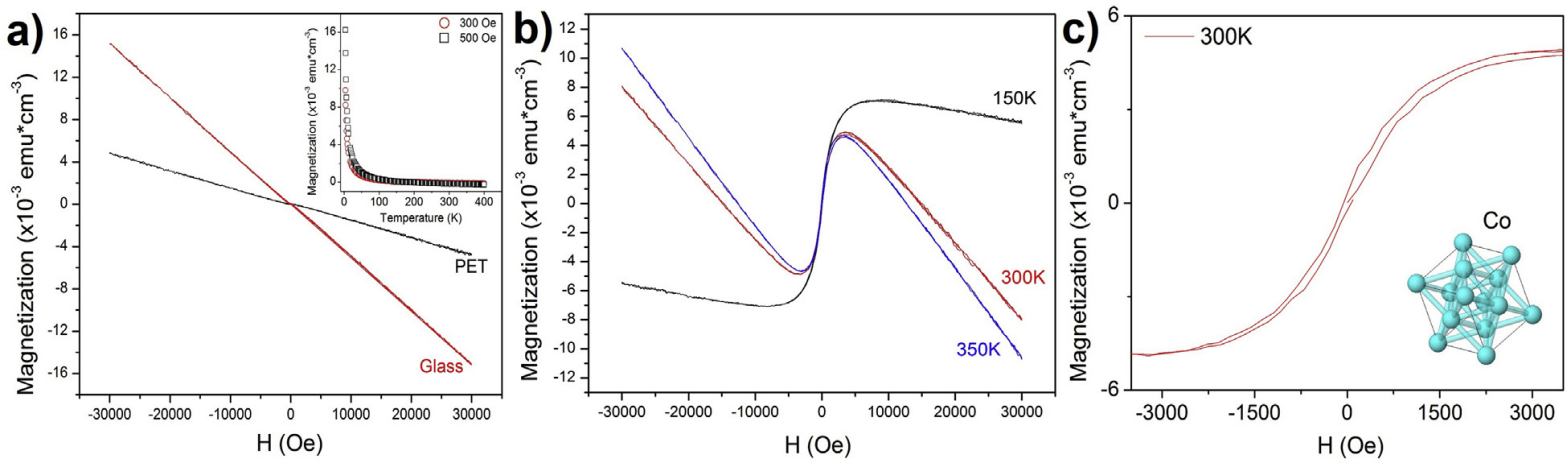
a) Magnetization behavior as a function of applied field of TiO_2_:Co thin films on ITO/PET and glass substrates at 300 K. The inset shows the dependence of magnetization with temperature for 300 and 500 Oe, b) M vs H for 150, 300 and 350 K for TiO_2_:Co thin films on glass with Ts = 293.5 K with annealing process and c) Magnetization as a function of applied magnetic field evidencing the hysteresis loop of TiO_2_:Co/glass sample. Inset shows the Co structure.

Figure 5a presents magnetization (M) behavior as a function of the applied field (H) at room temperature of TiO_2_:Co thin films on ITO/PET and glass substrates without annealing. It was possible to observe a diamagnetic-like tendency due to the substrate contribution; however, at low fields the magnetization measurements as a function of temperature (inset Figure 5a) shows at paramagnetic-like behavior of sample on glass substrate. This behavior was associated to the major contribution of species with permanent magnetic moments of the TiO_2_ semiconductor matrix.

**Figure 6:**
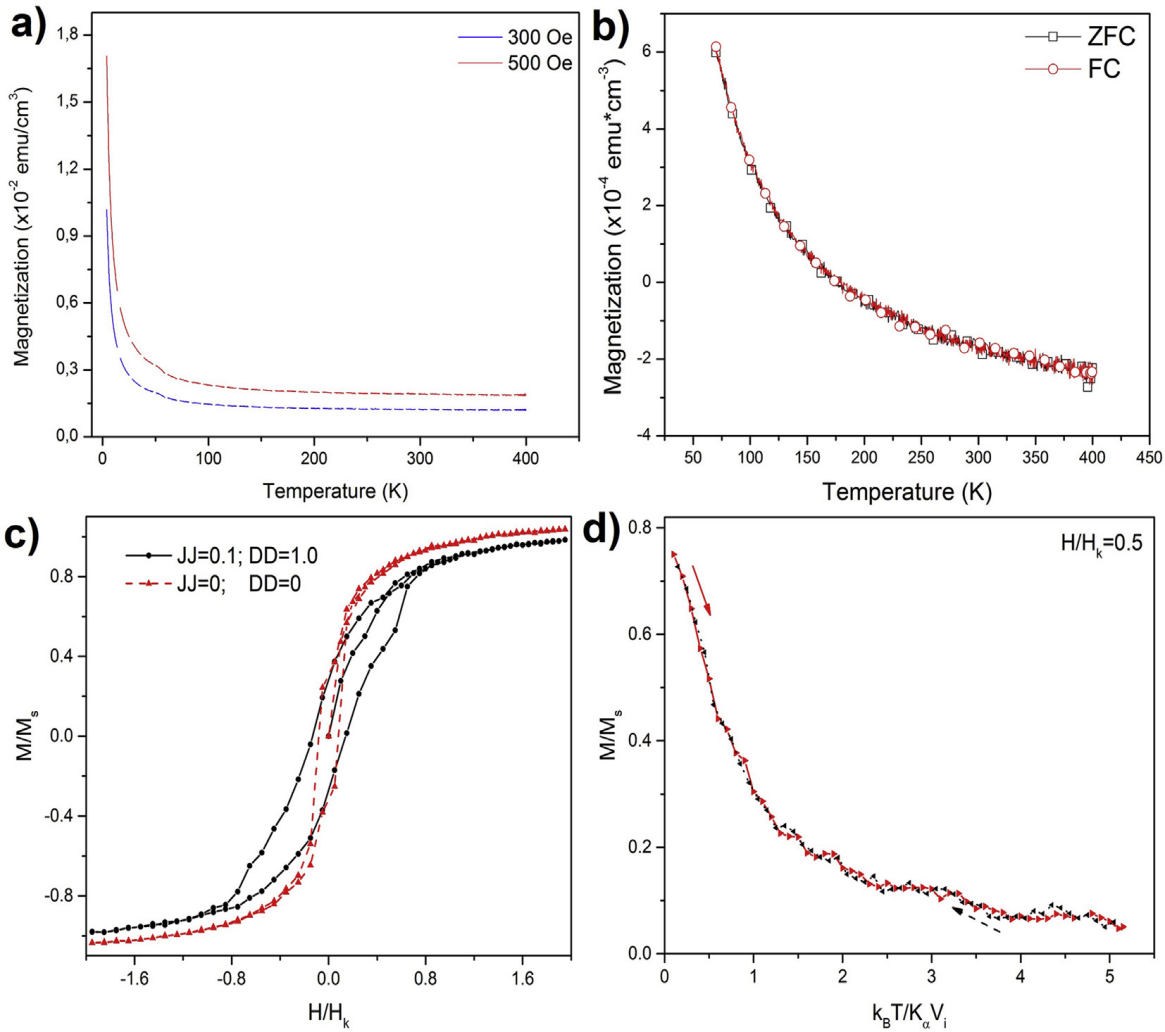
TiO_2_:Co/glass a) magnetization as a function of temperature for 300 and 500 Oe, b) ZFC and FCC magnetization curves for 500 Oe varying the temperature between 74 and 300 K. c) Dimensionless results of simulations of magnetization as a function of external field (where $H_{k}=\frac{K_{\mu }}{\mu_{0}M_{s}}$), and d) ZFC-FC simulated curves without dipolar (DD) and exchange (JJ) interactions. The simulated curves did not consider the substrate contributions.

In comparison, Figure 5b shows to different temperatures the magnetization curves as a function of applied magnetic field in annealing TiO_2_:Co/glass sample (Ts = 293.5 K and Ta = 473 K). The presence of hysteresis loop (Figure 5c) with a low coercive field (H_c_) evidence a ferromagnetic-like behavior. The non-presence of magnetic domains was corroborated by magnetization as a function of temperature and ZFC-FC measurements (Figures 6a and 6b), and hysteresis loop was related with a larger interaction among the Cobalt ions [22].

We considered a paramagnetic-matrix system with a high magnetic interaction associated to the Co ions randomly located in the TiO_2_ amorphous matrix [35, 36, 37]. This model agrees with the hysteresis loops and ZFC-FCC measurements (Figures 5, 6a, and 6b).

The contribution of dipolar interaction of the Co ions in matrix, can be evidence through a model based on a three-dimensional Heisenberg Hamiltonian described by five terms by each magnetic particle:

(1) $$H_{i}=-{\vec{\mu }}_{i}\cdot \vec{B}-K_{\alpha }V_{i}{\left(\frac{{\vec{\mu }}_{i}}{\left|{\vec{\mu }}_{i}\right|}\cdot {\vec{e}}_{i}\right)}^{2}+\mu_{0}J_{i}\underset{j}{\sum }{\vec{\mu }}_{j}\cdot {\vec{\mu }}_{i}-\frac{\mu_{0}}{4\pi }\underset{j}{\sum }\left(\frac{3\left({\vec{\mu }}_{i}\cdot {\vec{r}}_{ij}\right)\left({\vec{\mu }}_{j}\cdot {\vec{r}}_{ij}\right)}{{\left|{\vec{r}}_{ij}\right|}^{5}}-\frac{{\vec{\mu }}_{j}\cdot {\vec{\mu }}_{i}}{{\left|{\vec{r}}_{ij}\right|}^{3}}\right),$$

where the first term is Zeeman energy with $\vec{B}$ as the external applied magnetic field, $K_{\alpha }V_{i}{\left(\frac{{\vec{\mu }}_{i}}{\left|{\vec{\mu }}_{i}\right|}\cdot {\vec{e}}_{i}\right)}^{2}$ is the anisotropy energy where ${\vec{e}}_{i}$ is the vector along the magnetization easy axis of the magnetic atom (we assumed a random distribution of the easy magnetization directions of each Co dipole), and $K_{\alpha }$ is the anisotropy constant. The exchange energy term is $\mu_{0}J_{i}\underset{j}{\sum }{\vec{\mu }}_{j}\cdot {\vec{\mu }}_{i}$, where $\mu_{0}$ is the vacuum permeability and $J_{i}$ is the nearest-neighbor exchange-coupling constant between two ions, and the last term is the dipolar interaction with ${\vec{r}}_{ij}$ the distance vector of two dipoles [38]. The total energy of the system is determined by $H=\sum H_{i}$.

The concentration of magnetic atoms was obtained around of 12% by EDXS measurements, with a uniformly random distribution in a 11 × 11 × 11 lattice with periodic boundary conditions. For numerical propose, we define the dimensionless variables in terms of intrinsic saturation magnetization per particle, the anisotropy constant and the exchange coupling. Monte Carlo simulations with the standard Metropolis algorithm were used to calculate the matrix equilibrium magnetization after 1000 Monte Carlo steps (MCS), which averaged the dipolar magnetic moments every 100 MCS to avoid correlations among data.

Following that model, first all dipoles were oriented in the same direction of the external field and a random Euler matrix of rotation was applied in a point of the lattice. On the other hand, Metropolis criterion [39] accepted or rejected the new configuration, according to the probability of the Boltzmann distribution in a canonical ensemble. Magnetization is defined as $\frac{1}{V_{mag}}\langle \underset{i}{\sum }{\vec{\mu }}_{i}\cdot \frac{\vec{B}}{\left|\vec{B}\right|}\rangle$, where $V_{mag}$ corresponds to the total volume of all single domains and $\langle \underset{i}{\sum }{\vec{\mu }}_{i}\cdot \frac{\vec{B}}{\left|\vec{B}\right|}\rangle$ is the average value of the projection of total dipole moment.

Figure 6c shows the simulation of contributions to magnetization by the Zenner energy and anisotropy (red line) and the addition of the dipolar and exchange interactions (black line), where $M_{s}$ is the saturation magnetization of the system. For effects of the simulation, the dipolar and exchange interactions were normalized in $DD=\frac{VM_{s}}{4\pi d_{0}^{3}H_{k}}$ and $JJ=\frac{J_{ij}VM_{s}}{H_{k}}$ terms, respectively. The formation of a hysteresis loops in both cases with a low coercive field that increased with the dipolar and exchange interaction was obtained, according to experimental measurements of TiO_2_:Co thin films on glass substrates at 300 K (Figure 5c).

Figure 6d shows the ZFC-FCC simulated curves for this case, where only Zenner energy and anisotropy terms were considered to contribute to the system. This behavior evidences the ferromagnetic-like behavior due to a material with different magnetic interactions (Figure 6c). These results agree with experimental results and it is possible to associate the hysteresis loop of the TiO_2_:Co thin film to the dipolar interaction of the Co ions into the TiO_2_ matrix semiconductor and the high anisotropy of Co [40].

This result can be extrapolated to other oxide spintronic systems, where the magnetic impurities occupy a tetrahedral position. In these systems we expect than the magnetic interactions have the same functional form and only the constants and proportionalities change. In the case of Co-doped ZnO, magnetic impurities in tetrahedral positions were reported, and like TiO_2_:Co a room temperature ferromagnetism still being controversial [41, 42].

## 4 Conclusion

TiO_2_:Co thin films on flexible ITO/PET and glass substrates were fabricated via DC magnetron co-sputtering at room temperature. Structural characterization from XRD and μXRD measurements showed the formation of anatase and rutile phases, like semiconductor matrix. However, smaller Co crystals in the DMS matrix were founded in the TiO_2_:Co thin films on ITO/PET due to species mobility and thermal stabilization of the TiO_2_ structure in this substrate. The morphological properties showed formation of small grains on the surface associated to synthesis temperature and diffusion of the species. Magnetization as a function of temperature evidenced a paramagnetic-like behavior of the TiO_2_:Co thin films. The hysteresis loops at high temperature was associated to the dipolar interaction of Co ions in semiconductor matrix deposited on glass substrate. This large-range interaction was introduced in a theorical model, where simulated magnetization showed a hysteresis loops in the matrix doped with 12 wt% Co. Computational results confirmed the effect in the coercive field of dipolar interaction and anisotropy terms.

## Declarations

### Author contribution statement

Heiddy P. Quiroz: Conceived and designed the experiments; Performed the experiments; Analyzed and interpreted the data; Contributed reagents, materials, analysis tools or data; Wrote the paper.

E. F. Galíndez: Performed the experiments; Analyzed and interpreted the data; Wrote the paper.

A. Dussan: Conceived and designed the experiments; Performed the experiments; Analyzed and interpreted the data; Contributed reagents, materials, analysis tools or data; Wrote the paper.

### Funding statement

This work was supported by Universidad Nacional de Colombia - Quipu Code: 201010020958. Heiddy Paola Quiroz was supported by a PhD scholarship from of Doctorados COLCIENCIAS Conv. 727 - 2015.

### Competing interest statement

The authors declare no conflict of interest.

### Additional information

No additional information is available for this paper.
